# Supplementary material for: Economic disparity among generations under the Paris Agreement
Source: Nat Commun. 2021 Oct 5;12:5663. doi: 10.1038/s41467-021-25520-8 (PMC8492698; doi:10.1038/s41467-021-25520-8)
Supplement: Supplementary file 1 — Supplementary Information [file 41467_2021_25520_MOESM1_ESM.docx]

Supplementary information for

Economic disparity among generations under Paris Agreement

Haozhe Yang^1^, Sangwon Suh^1*^

^1^ Bren School of Environmental Science and Management, University of California, Santa Barbara, CA, USA

^*^ suh@bren.ucsb.edu

**Content**

1. Supplementary Note

2. Supplementary Tables 1-2

3. Supplementary Figures 1-5

**1.Supplementary Notes: Extended background on IAMs**

Here we classify the current economic modules of IAMs into three categories based on the literature^1^.

**1.1 General Equilibrium model**

Computational General Equilibrium (CGE) models are an algebraic representation of the intricate functioning of a market economy based on the economic equilibrium theory. By maximizing consumer utility and producer profits, the supply and demand reach an equilibrium in a CGE model. The general way that CGE models are used for policy analysis is to compute the new equilibrium after changing one or more variables affected by the policy to be analyzed (“shock”). Comparing the new counterfactual equilibrium to the initial equilibrium, including activity levels, prices and utility, provides insights about the effect of a “shock” on the economy.

IAMs using the CGE principles include: AIM/CGE, GEM, REMIND, WITCH and IMACLIM.

**1.2 Partial Equilibrium model**

Partial equilibrium analysis differs from general equilibrium modelling primarily by focusing on a specific market or sector. Partial equilibrium analysis is used extensively to estimate the impacts of climate change in different sectors of the economy.

IAMs using the partial equilibrium model include: GCAM, MESSAGE, TIAM-UCL.

**1.3 Energy system models**

Energy system models can broadly be classified as optimization models or

simulation models. Optimization models use information on costs and constraints of technology characteristics to identify the “best”, “least-cost” or “optimal” technology. The consumer is assumed to be rational, and energy supplies are allocated to energy demands, based on minimum lifecycle technology costs.

IAMs using the energy system models include: MESSAGE, DNE21+.

**2. Supplementary Tables**

**Supplementary Table 1. The ratio of the age-specific income versus the mean income at country level.**

| Country/Age | 0-17 | 18-25 | 26-40 | 41-50 | 51-65 | 65-75 | >75 |
| --- | --- | --- | --- | --- | --- | --- | --- |
| Australia | 0.91 | 1.14 | 1.06 | 1.05 | 1.11 | 0.83 | 0.64 |
| Austria | 0.87 | 1.06 | 0.93 | 1.07 | 1.14 | 0.95 | 0.92 |
| Belgium | 0.95 | 1.07 | 1.02 | 1.07 | 1.12 | 0.85 | 0.73 |
| Canada | 0.92 | 1.01 | 0.99 | 1.06 | 1.11 | 0.94 | 0.87 |
| Chile | 0.87 | 0.92 | 1.18 | 1.05 | 1.03 | 0.96 | 0.90 |
| Costa Rica | 0.77 | 0.98 | 1.10 | 1.11 | 1.15 | 1.03 | 0.92 |
| Czech Republic | 0.97 | 1.07 | 1.09 | 1.10 | 1.08 | 0.76 | 0.68 |
| Denmark | 1.00 | 0.85 | 0.96 | 1.12 | 1.20 | 0.87 | 0.73 |
| Estonia | 1.07 | 1.02 | 1.14 | 1.10 | 1.00 | 0.74 | 0.60 |
| Finland | 1.00 | 0.85 | 0.99 | 1.14 | 1.17 | 0.90 | 0.72 |
| France | 0.91 | 0.94 | 0.94 | 1.04 | 1.16 | 1.04 | 0.94 |
| Germany | 0.93 | 0.91 | 0.96 | 1.10 | 1.16 | 0.93 | 0.85 |
| Greece | 0.92 | 0.93 | 1.04 | 1.01 | 1.10 | 1.01 | 0.88 |
| Hungary | 0.93 | 1.03 | 1.06 | 1.06 | 1.01 | 0.94 | 0.91 |
| Iceland | 0.92 | 1.07 | 0.89 | 1.05 | 1.18 | 1.04 | 0.78 |
| Ireland | 0.94 | 1.10 | 1.03 | 1.04 | 1.12 | 0.86 | 0.75 |
| Israel | 0.87 | 0.99 | 0.99 | 1.09 | 1.23 | 1.11 | 0.92 |
| Italy | 0.86 | 0.99 | 0.96 | 0.99 | 1.17 | 1.08 | 0.92 |
| Japan | 0.95 | 1.06 | 1.01 | 1.10 | 1.16 | 0.90 | 0.85 |
| Korea | 1.00 | 1.11 | 1.06 | 1.09 | 1.07 | 0.73 | 0.56 |
| Latvia | 1.07 | 1.08 | 1.19 | 1.12 | 0.95 | 0.75 | 0.59 |
| Lithuania | 0.94 | 1.13 | 1.13 | 1.10 | 1.08 | 0.75 | 0.66 |
| Luxembourg | 0.90 | 0.89 | 0.98 | 1.06 | 1.07 | 1.11 | 1.02 |
| Mexico | 0.81 | 1.11 | 1.09 | 1.06 | 1.22 | 0.98 | 0.84 |
| Netherlands | 0.97 | 0.91 | 1.00 | 1.09 | 1.12 | 0.91 | 0.77 |
| New Zealand | 0.83 | 1.01 | 0.98 | 1.12 | 1.25 | 0.95 | 0.71 |
| Norway | 0.95 | 0.86 | 0.93 | 1.08 | 1.23 | 1.01 | 0.77 |
| Poland | 1.00 | 0.98 | 1.09 | 1.04 | 0.99 | 0.86 | 0.86 |
| Portugal | 0.94 | 0.96 | 1.03 | 1.00 | 1.05 | 1.07 | 0.90 |
| Slovak Republic | 0.87 | 1.04 | 1.07 | 1.05 | 1.08 | 0.89 | 0.82 |
| Slovenia | 0.98 | 1.09 | 1.03 | 1.07 | 1.03 | 0.89 | 0.80 |
| Spain | 0.92 | 0.99 | 0.95 | 1.02 | 1.13 | 1.02 | 0.88 |
| Sweden | 0.95 | 0.99 | 0.96 | 1.10 | 1.21 | 0.97 | 0.70 |
| Switzerland | 0.89 | 1.04 | 1.00 | 1.09 | 1.16 | 0.88 | 0.76 |
| Turkey | 0.84 | 0.98 | 1.10 | 1.11 | 1.14 | 0.89 | 0.81 |
| United Kingdom | 0.91 | 1.04 | 1.04 | 1.15 | 1.08 | 0.89 | 0.77 |
| United States | 0.87 | 0.93 | 1.02 | 1.09 | 1.16 | 1.02 | 0.81 |
| Brazil | 0.75 | 0.97 | 1.04 | 1.10 | 1.24 | 1.18 | 1.18 |
| Bulgaria | 0.94 | 1.04 | 1.15 | 1.18 | 1.07 | 0.73 | 0.59 |
| China (People's Republic of) | 0.85 | 1.06 | 1.14 | 1.03 | 1.06 | 0.85 | 0.82 |
| India | 0.84 | 1.04 | 1.03 | 1.15 | 1.15 | 1.07 | 1.12 |
| Romania | 0.90 | 0.97 | 1.13 | 1.09 | 1.06 | 0.87 | 0.75 |
| Russia | 0.87 | 1.01 | 1.07 | 1.09 | 1.07 | 0.83 | 0.79 |
| South Africa | 0.76 | 0.90 | 1.07 | 1.36 | 1.47 | 0.94 | 0.99 |
| Other | 0.85 | 1.01 | 1.07 | 1.10 | 1.07 | 0.87 | 0.82 |

**Supplementary Table 2. Regional relative cost of climate change mitigation versus global relative cost** ^2^**.**

The relative cost is computed as the cumulative costs of mitigation over the period 2020 – 2100 divided by cumulative GDP over that period.

| Region | Median | 25% percentile | 75% percentile |
| --- | --- | --- | --- |
| OECD 1990 | 0.47 | 0.41 | 0.61 |
| Asia | 1.47 | 1.19 | 1.64 |
| Middle East and Africa | 2.29 | 1.79 | 2.79 |
| Latin America | 0.99 | 0.92 | 1.15 |
| Economies in Transition | 1.99 | 1.39 | 2.49 |

**Supplementary Figures**

**
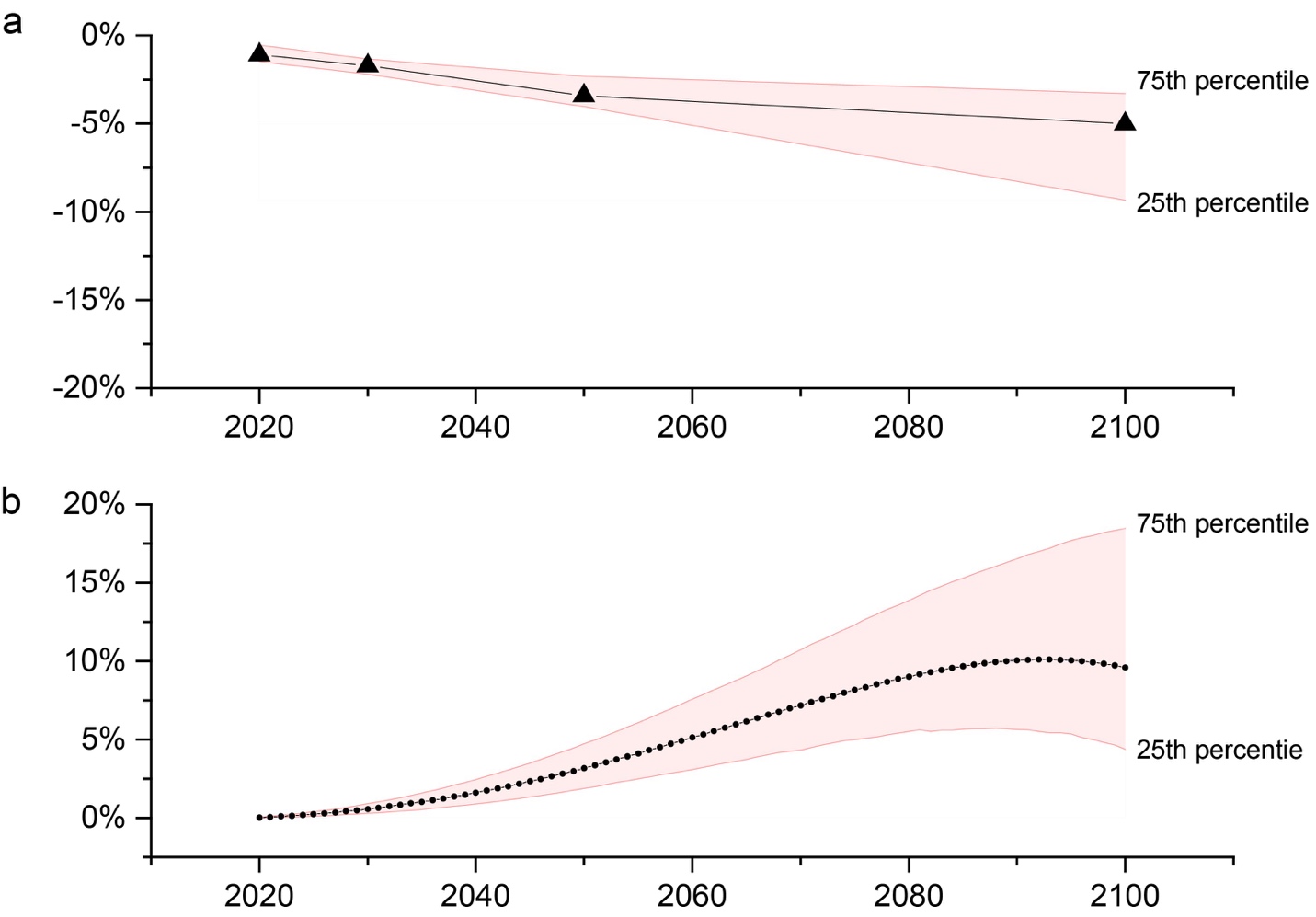
**

**Supplementary Figure 1. Time series of the costs and benefits from 2020 – 2100.**

**a** Global cost of climate change mitigation relative to global GDP. **b** Global benefits of climate change relative to global GDP. Data are presented as median values with 25% and 75% percentile, n=17 climate models in (**a**) and n=1000 replicates in (**b**).

**
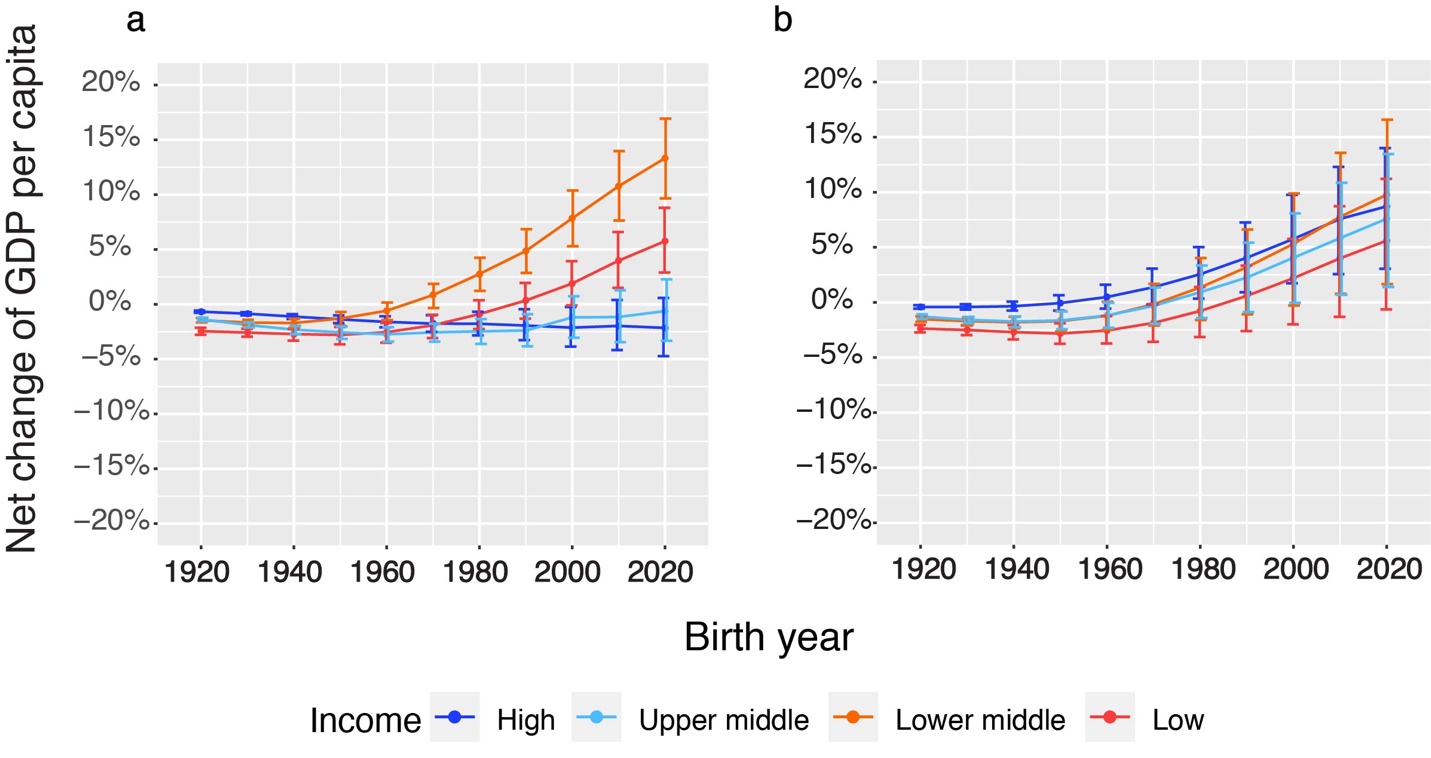
**

**Supplementary Figure 2. The uncertainty of the population-weighted change in GDP per capita by income group when using different model specifications to evaluate the effects that temperature has on GDP growth.**

The population-weighted average of the percentage change in GDP per capita by income group using **a** short-term BHM damage function, **b** long-term (5-year lag) BHM damage function.

The data are presented as median values, and the error bar represents the 25% and 75% percentile level, n=47 high-income countries, n=50 upper-middle-income countries, n=43 lower-middle-income countries and n=29 low-income countries.


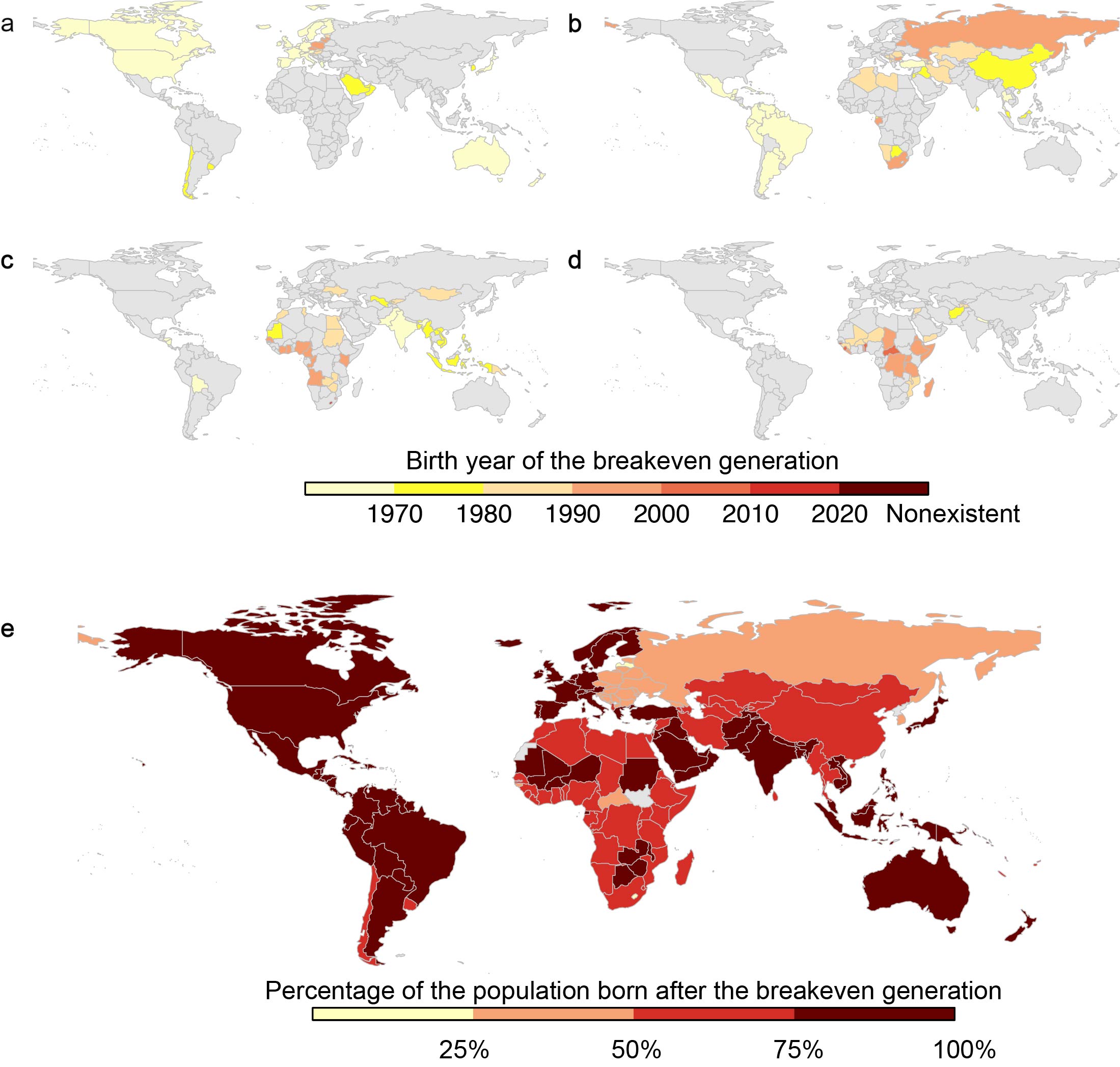


**Supplementary Figure 3. Breakeven generation and the percentage of population born after the breakeven generation using the long-term net benefits.**

The birth year of the breakeven generation in 2020 in **a** high-income countries, **b** upper-middle-income countries, **c** lower-middle-income countries and **d** low-income countries. **e** The percentage of population born after the breakeven generation. In (**a**) – (**d**), different colors represent different ranges for the birth years. In (**e**), different colors represent different ranges for the percentage of the population. Here, we use the long-term benefits of climate change mitigation to measure the benefits of climate change mitigation.


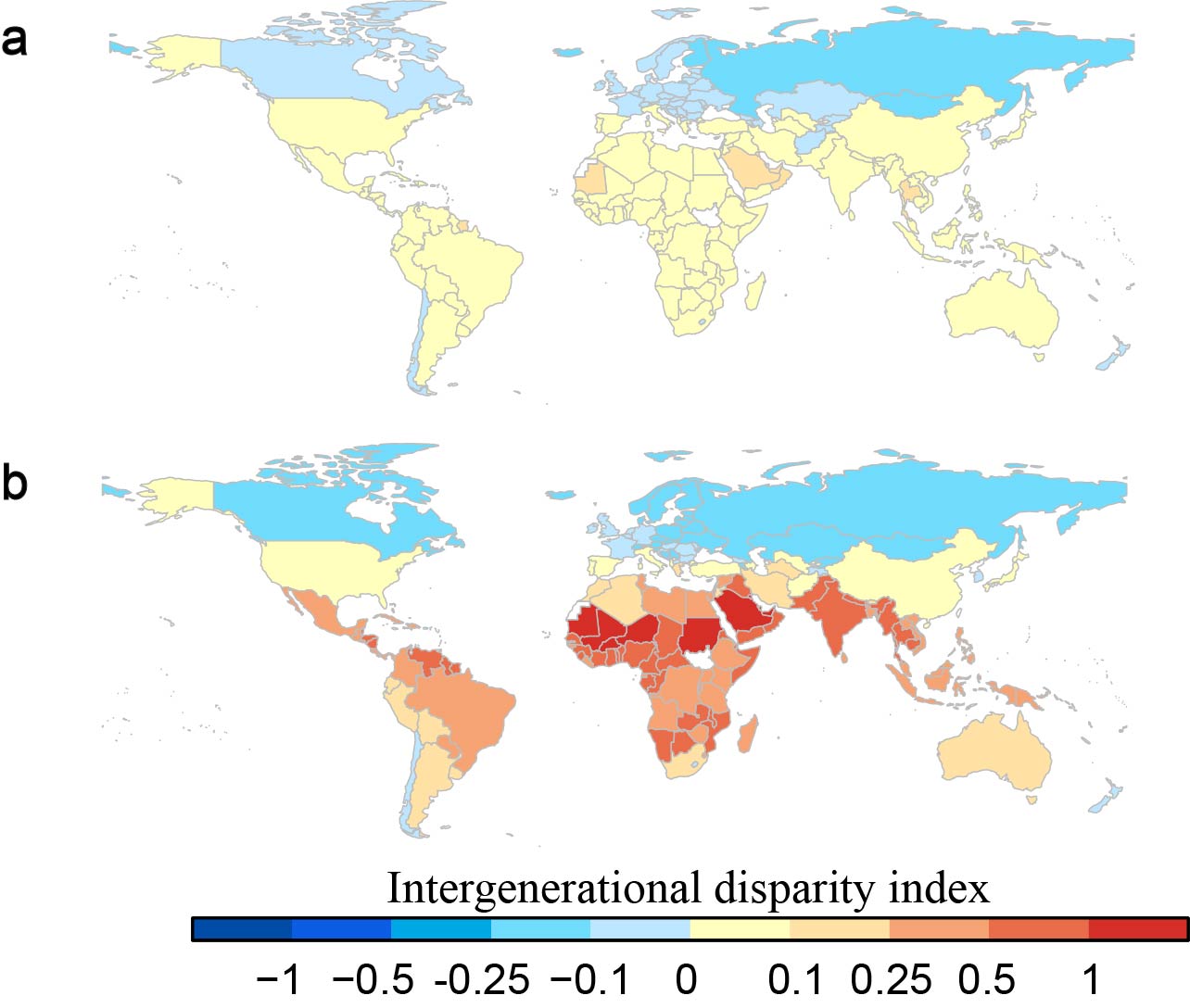


**Supplementary Figure 4. The intergeneration disparity index using short-term net benefits of climate change mitigation.**

The intergeneration disparity index using short-term net benefits of climate change mitigation

in **a** 2020 and **b** 2100.


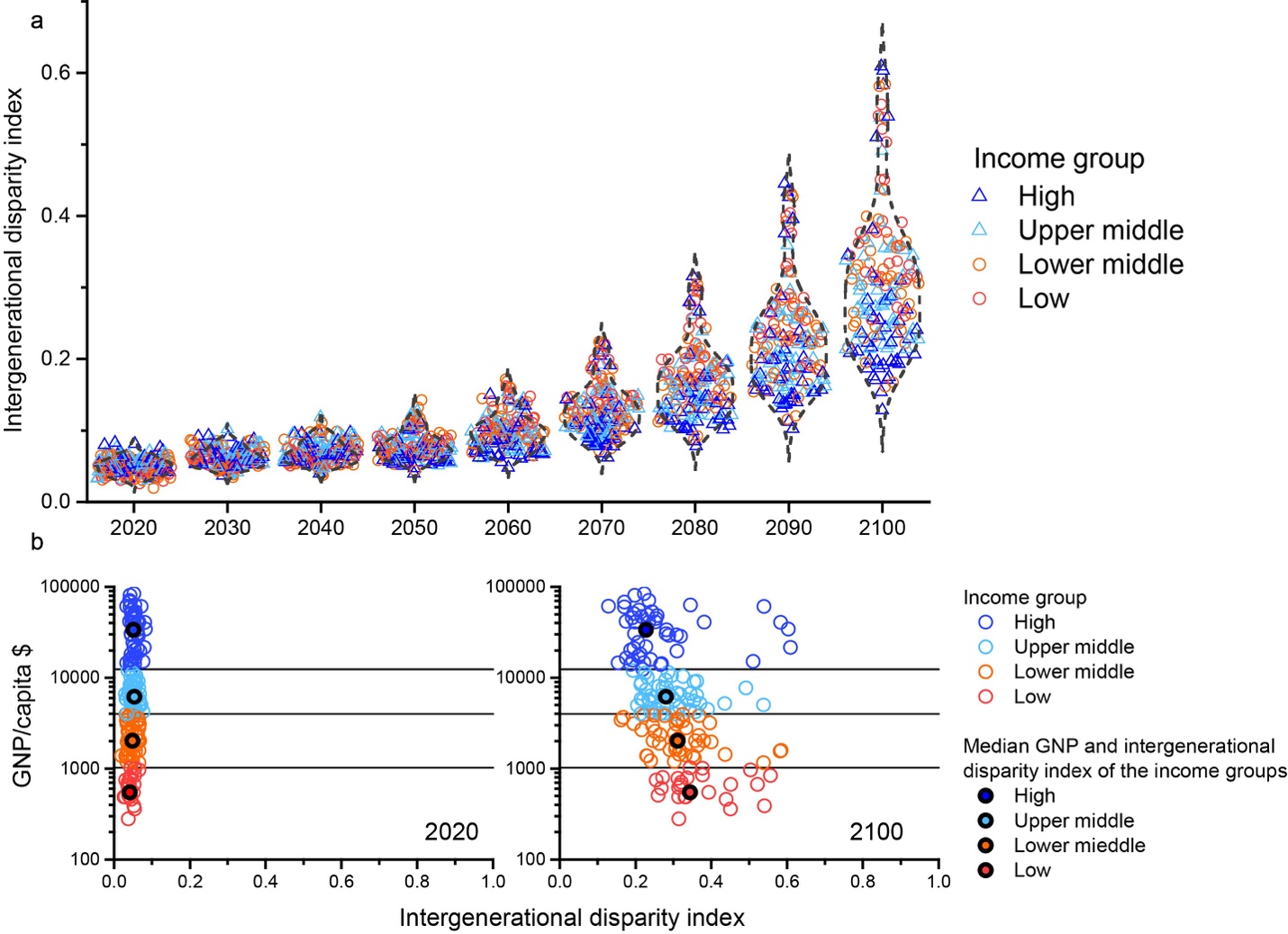


**Supplementary Figure 5. Intergenerational disparity among high-, upper-middle-, lower-middle- and low-income countries using the long-term net benefits.**

**a** The distribution of the intergenerational disparity index from 2020 to 2100. **b** Intergenerational disparity index and GNP (gross national product) per capita (using 2018 GNP per capita) in 2020 and 2100. In (**a**), a triangle symbol represents a higher income country, and a circle symbol represents a lower income country. In (**b**), a circle symbol represents a country, and a solid circle with the black edge represents the median GNP and the median intergenerational disparity index of an income group. In (**a**) and (**b**), the color of a symbol represents the income group of a country. The intergenerational disparity index is calculated as the percentage change in lifetime GDP per capita among the 25-year-old age cohort minus that among the 75-year-old age cohort.

Here, the intergenerational index is calculated by assuming the long-term net benefits of climate change mitigation.

**References**

1. Nikas, A., Doukas, H. & Papandreou, A. A Detailed Overview and Consistent Classification of Climate-Economy Models. *Understanding Risks and Uncertainties in Energy and Climate Policy: Multidisciplinary Methods and Tools for a Low Carbon Society* 1–54 (2019).

2. IPCC. *Climate Change 2014: Mitigation of Climate Change. Contribution of Working Group III to the Fifth Assessment Report of the Intergovernmental Panel on Climate Change*. vol. 6 (Cambridge University Press, 2014).
